# Some reflections on symmetry: pitfalls of automation and some illustrative examples

**DOI:** 10.1107/S2056989019014907

**Published:** 2019-11-08

**Authors:** William Clegg

**Affiliations:** a Chemistry, School of Natural and Environmental Sciences, Newcastle University, Newcastle upon Tyne NE1 7RU, England

**Keywords:** symmetry, pseudo-symmetry, twinning, disorder, chirality, structure validation, crystal structure

## Abstract

In the context of increasing hardware and software automation in the process of crystal structure determination by X-ray diffraction, and based on conference sessions presenting some of the experience of senior crystallographers for the benefit of younger colleagues, an outline is given here of some basic concepts and applications of symmetry in crystallography. Three specific examples of structure determinations are discussed, for which an understanding of these aspects of symmetry avoids mistakes that can readily be made by reliance on automatic procedures. Topics addressed include pseudo-symmetry, twinning, real and apparent disorder, chirality, and structure validation.

## Abstract 2

An outline is given of some basic concepts and applications of symmetry in crystallography. Three specific examples of structure determinations are discussed, for which an understanding of these aspects of symmetry avoids mistakes that can readily be made by reliance on automatic procedures. Topics addressed include pseudo-symmetry, twinning, real and apparent disorder, chirality, and structure validation.

## Introduction   

Automation of both hardware and software plays an increasingly significant role in many areas of science. Some aspects of crystallography, especially the determination of crystal structures from diffraction data, lend themselves readily to automated procedures. Automation of hardware under computer control is particularly prevalent at central research facilities providing powder and single-crystal diffraction beamlines and instruments using both neutrons and synchrotron X-rays. This includes feedback control of beamline optics and other hardware, the monitoring and control of sample environments, and robotic exchange of samples (which may themselves, especially for protein samples, have been crystallized by robots). Software automation covers all stages of the diffraction experiment, data processing, structure solution and refinement, and the analysis and presentation of results. There has long been a desire among many commercial suppliers and the users of their equipment and programs for a fully automatic ‘GO Button’ approach to structure determination and this has been essentially realized in some integrated software packages.

Automation brings both advantages and pitfalls. On the positive side, it usually leads to a significant increase in throughput and productivity, ensures that procedural steps are not omitted, and should avoid the inadvertent loss of primary data and metadata generated in the experiment and subsequent processing. In straightforward cases all runs smoothly and a reliable result is achieved. One of the supposed selling points of crystal-structure determination software automation is that it removes the need for crystallographic expertise, such that a research chemist with little or no crystallographic training can use an automated system to obtain a structure for a newly synthesized compound. On the negative side, we can use the same words: it removes the need for crystallographic expertise, which means that potential problems may lie undiscovered, the new structure being in some way defective or misleading, or the automatic procedures used being inappropriate for the underlying structural questions being asked. Non-routine issues such as twinning, structural disorder, and modulated structures (both commensurate and incommens­urate) are often poorly treated, or completely overlooked, by automatic software, and require individual personal attention by an expert.

One consequence of increasing automation in crystallography is the dilution or even the loss of knowledge and understanding of fundamental principles and key practices that have been generated in the past and are now being learnt and applied by fewer scientists, including those whose specific responsibility is the provision of a crystal-structure determ­ination service in academia or industry. The organization and delivery of major national and inter­national training courses helps to counter this sad development, but these are demanding in time and financial cost and are often oversubscribed. We are heavily reliant on the personal crystallographic training of young scientists by expert research group leaders, and on the support of IUCr and its regional and national affiliates.

Aspects of this erosion of crystallographic expertise and the contribution of automation to it are occasionally addressed by specific conference sessions. The ACA annual meeting in Denver in 2016 included a session with the provocative title ‘Things we no longer need to know’ and the 2019 ECM in Vienna had a microsymposium, jointly organized by the Education and Senior Crystallographers’ General Inter­est Groups of the ECA, whimsically entitled ‘Teaching new dogs old tricks’. This article is based on material presented at those two meetings, focusing on the particular topic of symmetry. A general introduction and a review of key concepts and terms related to crystallographic symmetry are followed by a selection of examples that illustrate the value of experience and expertise in avoiding significant errors in crystal-structure determination.

Many chemistry students learn a smattering of symmetry in their undergraduate courses. This is most often restricted largely to point-group symmetry as applied to individual mol­ecules, and may include a recipe-driven application of rules derived from group theory for some understanding of aspects of chemical bonding and spectroscopy, such as the number of fundamental vibrations and their expected appearance in infrared and Raman spectra, and in the application of symmetry restrictions on certain kinds of reaction mechanisms. Any knowledge of space-group symmetry is probably very sketchy, and the role of symmetry in crystallography may be little more than a list of crystal systems.

When chemists (and other scientists) with such rudimentary understanding of symmetry encounter crystallography in real research, they rely almost entirely on automatic procedures and accept the output of these without question. It is widely believed that a deeper knowledge of symmetry is unnecessary, because these issues are handled by the software. The crystal system is chosen by the diffractometer control system. The space group is determined automatically (and apparently unambiguously) during or after data collection. In any case, the space group need not be established before attempting to solve the structure, because modern programs such as *SUPERFLIP* (charge flipping; Oszlányi & Sütő, 2004 ▸; Palatinus & Chapuis, 2007 ▸) and *SHELXT* (Sheldrick, 2015*a* ▸) work it out as part of solving the structure, automatically identifying symmetry elements from the derived electron density or reflection phase relationships. Symmetry-imposed constraints on atomic positional and displacement parameters are dealt with by refinement programs without much comment, as are any symmetry-equivalent atoms needed for full mol­ecular geometry calculations when mol­ecules have crystallographic symmetry (*Z′* < 1) or are polymeric. Popular graphics programs also handle mol­ecular and space-group symmetry automatically. If any mistakes are made regarding symmetry, we can trust validation programs such as *PLATON* (Spek, 2003 ▸) and *checkCIF* (Spek, 2009 ▸) to let us know and, in some cases, correct the error for us.

## A tutorial review of some important symmetry concepts and terms   

A full treatment of symmetry in crystallography is beyond the scope of this article. It is a fundamental part of any systematic instruction in the subject and can be found in any significant relevant textbook as well as in the material of intensive training courses in crystal structure determination. Here we focus on a few key terms, some of which are often confused, misunderstood, and wrongly used.

First it is important to distinguish between *symmetry elements* and *symmetry operations*. In simple terms relevant to our subject, a *symmetry element* is a physically identifiable point, line, or plane in a mol­ecule or crystal structure about which symmetry operations are applied, while a *symmetry operation* is the act of inversion though a point, rotation about a line, or reflection in a plane (or a special combination of rotation with either inversion or reflection) that leaves the mol­ecule or structure afterwards with an identical appearance. Note that a pure rotation operation is actually achievable with a physical model by just turning it round, while the others are not and can be applied only in a graphical model representation, as they involve ‘turning the object inside-out’; this distinction divides symmetry operations into two sets: ‘proper rotations’ and ‘improper rotations’, of which inversion and reflection are special cases. For completeness, so that symmetry can be usefully treated by mathematical group theory, the list of symmetry operations for a mol­ecule or structure must include the so-called identity operation, which means leaving the object completely unchanged (‘doing nothing’, also a proper operation).

Each symmetry element present in a mol­ecule or structure provides one or more possible symmetry operations; for example, a threefold rotation axis provides operations of 120° rotation, 240° (or −120°) rotation, and 360° rotation (which is equivalent to the identity operation).

A *point group* is the total collection of all unique symmetry operations for a finite single object such as a mol­ecule. We refer here to symmetry operations rather than symmetry elements because a point group can then be treated by mathematical group theory; in crystallography we make use of various consequences of this. It should be noted that all symmetry elements pass through one point (in some point groups, a whole line or plane), which remains unmoved by any symmetry operation. Repeated use of any symmetry operation eventually returns the mol­ecule to the original orientation exactly, not just an equivalent form with identical appearance but with some atoms exchanged. Only certain combinations of symmetry elements are possible: for example, there can be 0, 1, or 3 fourfold rotation axes, but not 2; there cannot be more than one inversion centre; a twofold rotation axis must lie either in or perpendicular to a single mirror (reflection) plane if both are present, and in the latter arrangement their point of inter­section is an inversion centre.

Correspondingly, a *space group* is the total collection of all symmetry operations for a three-dimensional repeat pattern such as a crystal structure. [At this point it should be clarified that the strictly correct term here is *space-group type* rather than space group (Nespolo *et al.*, 2018 ▸); a full space-group specification includes also the unit-cell parameters for a particular structure. However, the use of space group instead of space-group type is widespread and we will continue to use it here in this sense for simplicity – any reader whose sense of propriety is offended by this can apply an automatic translation at every occurrence hereafter.] Because a crystal structure incorporates pure translation symmetry in its lattice, additional types of symmetry element and symmetry operation are possible here but not in individual mol­ecules: the combination of rotation with translation leads to screw axes, and the combination of reflection with translation leads to glide planes. For a space group, symmetry elements of the same kind are arranged parallel at regular inter­vals of half a unit-cell edge; they do not all pass through a single point. Repeated use of any symmetry operation on a chosen point in the structure may eventually return it to the original point, or take it to an equivalent point in a different unit cell, related to the original point by pure translation. Rotations (proper and improper, including screw axes) may be only of order 1, 2, 3, 4, or 6; other orders of rotation (5 and >6) are incompatible with a three-dimensional lattice. As for point groups, symmetry elements and operations can be combined only in certain ways. The total number of possible combinations is 230. Applying the same restrictions to the order of rotations in single finite objects, the total number of point groups related to crystal structures is also finite, at 32 (these point groups are also known as crystal classes).

Any crystal structure has, or belongs to, a specific space group. The choice of origin for the coordinate system describing positions in a crystal structure is, in principle, arbitrary, although there are conventions about how it should relate to the symmetry elements; it can legitimately be shifted to an equivalent point in any other unit cell. A diffraction pattern obtained from this crystal structure, however, has a central point corresponding to the (unmeasured) reflection with indices 0,0,0, so the symmetry of the diffraction pattern is expressed in terms of a point group, not a space group. Each space group thus has an associated point group, in which screws are reduced to simple rotations and glides are reduced to simple reflections.

There are thus, in principle, 32 possible point-group symmetries displayed by diffraction patterns. A centrosymmetric crystal structure gives a fully centrosymmetric diffraction pattern. In the absence of resonant scattering by any of the elements present in the structure (which is wavelength-dependent), the diffraction pattern of a non-centrosymmetric structure will also be centrosymmetric; this is known as Friedel’s Law [*I*(*h*,*k*,*l*) = *I*(−*h*,−*k*,−*l*)]. Significant resonant scattering (also known as anomalous scattering or anomalous dispersion) in a non-centrosymmetric crystal structure leads to a contravention of Friedel’s Law and a non-centrosymmetric point group for the diffraction pattern. Otherwise, the point-group symmetry of the diffraction pattern must be one of the centrosymmetric point groups, of which there are 11. These are known as the 11 *Laue classes* (not, strictly speaking, Laue groups). Thus each space group, of which there are 230, has a corresponding point group (32) and a corresponding Laue class (11). Observation of the Laue class by analysis of diffraction-pattern symmetry (effective equality or otherwise of possibly symmetry-equivalent reflections) is part of the process of deciding the correct space group for a crystal being examined by diffraction.

We can summarize the incidence of key symmetry terms in crystallography, and their relationships, in this way.

The *metric symmetry* is the symmetry of the unit-cell shape, ignoring the actual contents of the structure; there are six possible shapes (the same shape applies to hexa­gonal and trigonal structures), seven including the primitive rhombohedral unit cell, and these are related to, but do not define, the seven crystal systems – the definition is based on symmetry, not on geometrical shape, which is a consequence of the symmetry.

The *Laue symmetry* is the symmetry of the diffraction pattern, assuming Friedel’s Law; there are 11 Laue classes, 32 possible point groups if Friedel’s Law does not apply.

The *space-group symmetry* is the symmetry of the complete crystal structure, with 230 possibilities, which are very far from equally common in practice.

A *point group* is the symmetry of a single finite object; it has relevance in crystallography to metric symmetry, Laue classes, and well-formed crystal shapes, all of which can be described in terms of point groups. The environment of a mol­ecule in a crystal structure, and the shape of the mol­ecule itself, can also be described by point-group symmetry.

Any atom or mol­ecule lying on a pure rotation axis, a mirror plane, or an inversion centre in a crystal structure is said to be in a *special position*; application of these symmetry operations leaves the atom/mol­ecule in the same place, though some symmetry-equivalent atoms of a mol­ecule may be exchanged; the point-group symmetry is defined by the symmetry elements that inter­sect at that point.

Any point that is not on a pure rotation axis, mirror plane or inversion centre is a *general position*, and its point-group site symmetry is 1; all symmetry operations transform it to a different but symmetry-equivalent position in the structure.

Finally, the symmetry-independent part of a crystal structure, the full contents of which must be specified in order to characterize the entire structure, is called the *asymmetric unit*. It is a rational fraction of the unit-cell contents, and it is related to other parts of the unit cell (and to parts of other unit cells) by space group symmetry operations. The asymmetric unit of a crystal structure may consist of one mol­ecule (*Z′* = 1), more than one mol­ecule (*Z′* > 1), or a fraction of a mol­ecule that displays crystallographic symmetry within itself (*Z′* < 1).

## A case of pseudo-symmetry with added complications from twinning   

This first example is an organic compound with elements C, H, N, O, F, and S. Details of the material and the crystal structure cannot be given here because it was commercial research subject to a non-disclosure agreement. Diffraction data were collected at 150 K on an Oxford Diffraction Gemini A Ultra diffractometer with Cu *K*α radiation from a single crystal with maximum dimension 0.3 mm and of moderate visual quality.

The unit cell was identified by diffractometer control software as triclinic, with angles 116.348 (3), 90.082 (2), and 90.161 (2)°; probable space group *P* 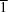, with *Z* = 4, *Z′* = 2. Recognizing that standard uncertainties on cell parameters from single-crystal diffractometers are generally reputed to be underestimated, the question arises whether this is really a monoclinic structure (with **a** as the monoclinic symmetry axis in this setting) with two 90° angles. There is, at least, metric pseudo-symmetry here, and we must examine the Laue symmetry for clearer evidence.

The diffractometer software *CrysAlis PRO* (Rigaku Oxford Diffraction, 2015 ▸) and the data processing program *XPREP* (Bruker, 2014 ▸) both prefer triclinic *P* 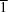 to any monoclinic option, based on the refined cell parameters with their standard uncertainties, *R*_int_ (assessing the agreement of intensities of equivalent reflections in each crystal system), and intensity statistics (an indication of the probability of inversion symmetry in the structure by comparison with centric and acentric intensity distributions); however, monoclinic *P*2_1_/*n* is also offered as a feasible option because two unit-cell angles are close to 90°, *R*_int_ for monoclinic Laue symmetry is 0.132 (a rather poor value, but not unknown for samples of this kind), and reflections *h*0*l* with *h* + *l* odd (after axis reassignment to give the conventional unique *b*-axis setting) have an average intensity approximately 10% that of general reflections, potentially representing glide plane systematic absences. Visual inspection of reciprocal lattice layers of the diffraction pattern perpendicular to the possible monoclinic symmetry axis gives a convincing impression of twofold rotation symmetry.

The structure can be readily solved separately in both triclinic and monoclinic settings by the program *SHELXT* (Sheldrick, 2015*a* ▸), which requires a prior choice of Laue class but not of space group, and the two solutions can be refined with essentially the same minor disorder in each case, to give structures in *P* 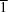 (*Z*′ = 2) and in *P*2_1_/*n* (*Z*′ = 1). Some key features and indicators of the two refinements, using *SHELXL* (Sheldrick, 2015*b* ▸), are given in Table 1 ▸.

While the triclinic setting is clearly preferred on the basis of Laue symmetry (*R*_int_), the refinement *R* factors are more ambiguous, as lower values are expected when more parameters are refined in a lower-symmetry model, and the monoclinic setting gives a cleaner difference map. So, with conflicting evidence, which is the correct solution?

The clue to the answer is given by the large mean observed/calculated intensity ratios *K* in the analysis of variance following refinement. These, together with the metric pseudo-symmetry of a triclinic lattice closely approximating a monoclinic one, are an indication of possible twinning of a type commonly known as pseudo-merohedral (Parsons, 2003 ▸) or twinning by pseudomerohedry (Nespolo & Ferraris, 2004 ▸). A twin law with matrix (1 0 0, 0 −1 0, 0 0 −1) represents a twofold rotation about the triclinic **a** axis. Because of the closeness of two unit cell angles to 90°, the two twin components related by this rotation give almost exact overlap, with near-coincidence of their reciprocal lattice points. Most of the observed reflections are thus a combination of two symmetry-inequivalent reflections from the two twin components.

Incorporation into the refinement of the twin law and a twin fraction (relative contributions of the two twin components) significantly improves the result for the triclinic model, so that it is now clearly the preferred solution. The second twin component has a fraction of almost 17%, so the two components are approximately in a 5:1 ratio. Relevant information is given in Table 2 ▸ (definitions are as for Table 1 ▸). The largest difference peak lies within a disordered di­methyl­sulfoxide solvent mol­ecule, which is partially modelled by two alternative positions of its sulfur atom (the lighter atoms cannot be satisfactorily resolved because of the low occupancy of the minor component).

**Figure 1:**
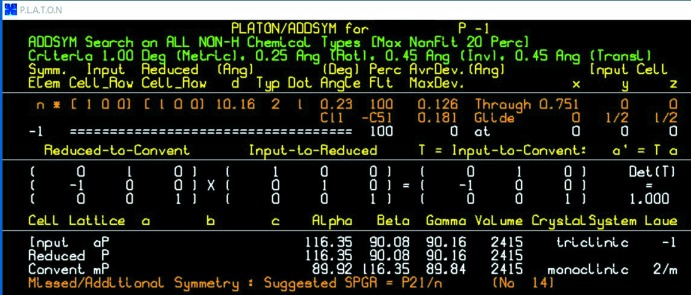
*PLATON* ADDSYM output for the first example.

This would appear to be a definitive result; expert opinion, at least from this author, prefers the twinned triclinic structure on virtually all criteria. It remains, however, an issue not entirely resolved by automatic procedures. The ADDSYM routine of *PLATON* (Spek, 2003 ▸) indicates 100% fit of the non-hydrogen atom positions to *n*-glide symmetry within default tolerances and suggests the monoclinic space group *P*2_1_/*n* (Fig. 1 ▸). The pseudo-symmetry is clearly very close!

## Disordered centrosymmetric or ordered non-centrosymmetric?   

This is not an uncommon question, and the immediate answer is ‘it all depends…’. Several factors need to be considered when a structure can be refined alternatively as disordered in a centrosymmetric space group or, at least apparently, ordered in a non-centrosymmetric space group. Important evidence comes not only from refinement indicators such as *R*-factors, satisfactory convergence, and difference map features, but also from the resultant mol­ecular geometry, including abnormal distortions and the need to impose refinement constraints and/or unusually strong restraints. There may also be relevant non-crystallographic data, for example information from the chemical synthesis method, spectroscopy, or physical properties. The example given here, already published from an academic research project (Clegg *et al.*, 1998 ▸), is typical in our experience of many compounds encountered in commercially sponsored pharmaceutical research.

**Figure 2:**
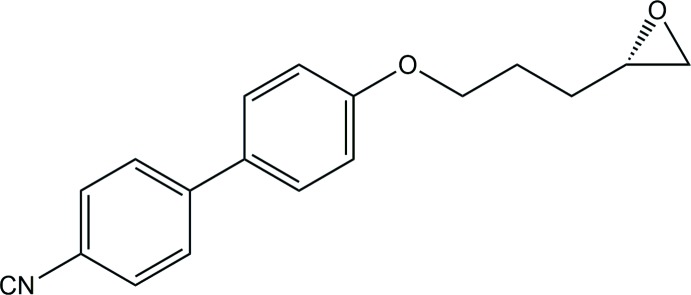
Chemical structure of the second example, an oxirane.

The ep­oxy or oxirane compound shown in Fig. 2 ▸ was studied during early commissioning of the then new synchrotron single-crystal diffraction facility of Station 9.8 at Daresbury Laboratory’s Synchrotron Radiation Source (SRS) in 1997 (Cernik *et al.*, 1997 ▸); prior investigation gave unusably weak diffraction with even the most powerful laboratory X-ray sources available at that time.

A monoclinic unit cell was readily found on a first-generation Bruker SMART 1K diffractometer/detector system, with data collected at 160 K. This work predated charge flipping and *SHELXT* and could not make use of the level of automation available now. The data reduction program *XPREP* supported monoclinic Laue symmetry on the basis of *R*_int_. Systematic absences clearly indicated a 2_1_ screw axis and suggested an *n*-glide plane, with relevant ‘absent’ reflections having an average intensity approximately 10% of the rest of the data. The program’s Figure of Merit ranking gave *P*2_1_/*n* as the preferred space group; this came above *P*2_1_, because the mean value of |*E* ^2^ − 1| was very close to that typical for a centric intensity distribution, indicating likely inversion symmetry in the crystal structure, and above *P*2_1_/*m* and *P*2/*m* because of the systematic absences and the relative scarcity of these space groups in the Cambridge Structural Database (Groom *et al.*, 2016 ▸). For *P*2_1_/*n* the asymmetric unit is one mol­ecule.

*SHELXS* (Sheldrick, 2008 ▸) with default parameters gives a clear solution, but the three-membered oxirane ring appears disordered. Refinement with *SHELXL* proceeds smoothly, with no unusual features except the ring disorder, and a conventional *R* factor of around 0.07 is obtained.

Running *SHELXS* with the assumption of space group *P*2_1_ and default parameters gives a less clear solution, with *Z*′ = 2 and a rather poorly defined and disordered oxirane ring in each of the two independent mol­ecules. From this a sensible ordered structural model can be derived by manual selection of peaks from the double images of the disorder, such that the ring has different orientations in the two mol­ecules – a slightly time-consuming but straightforward task given a basic understanding of organic mol­ecular geometry. This model leads to excellent refinement in *SHELXL*, with no reappearance of the manually removed disorder; all hydrogen atoms can be seen in a difference map, and the conventional *R* factor is 0.046 at convergence. All other refinement indicators are perfectly acceptable, and there are no significant features in a final difference map.

In this particular case (though by no means always!) the ordered non-centrosymmetric model is definitely correct. We know this because, although it was not explicitly stated at the time the sample was provided, the mol­ecule is known (or at least believed) to be chiral (as shown in Fig. 2 ▸) and enanti­o­pure from the synthesis, so a Sohncke (or Sohnke; both spellings are found) space group is necessary and a centrosymmetric space group is ruled out. There is insufficient resonant scattering from the light atoms in this structure for the absolute configuration to be confirmed from the diffraction experiment, and the correct enanti­omer is selected from the synthesis information. It is, however, clear that both mol­ecules have the same absolute configuration.

**Figure 3:**
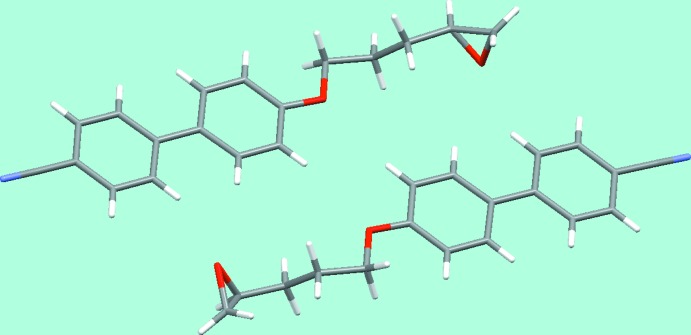
The asymmetric unit (two mol­ecules) of the oxirane.

**Figure 4:**
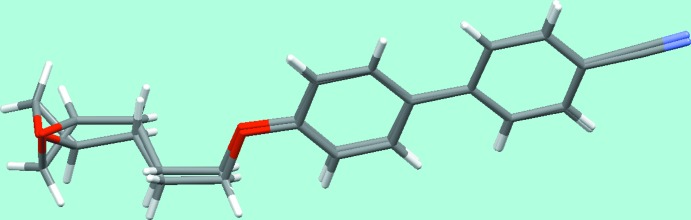
One mol­ecule of the asymmetric unit overlaid with best fit on an inverted form of the other mol­ecule.

The asymmetric unit is shown in Fig. 3 ▸, and a *Mercury*-generated overlay (Macrae *et al.*, 2008 ▸) of one mol­ecule and an inverted form of the other in Fig. 4 ▸. The two mol­ecules are almost completely related by a non-crystallographic inversion centre, only the common chirality of the two oxirane rings breaking the pseudo-inversion symmetry. This is the reason why the structure can readily be solved and refined in an incorrect centrosymmetric space group with disorder to model the superposition of the two rings. Both the inversion centre and the *n*-glide plane are only approximate, the latter giving rise to systematically weak reflections *h*0*l* with *h* + *l* odd. The compound is thus a pseudo-racemate, a situation we have found frequently for enanti­opure pharmaceutical and related compounds having just one chiral centre and two mol­ecules in the asymmetric unit.

How does more modern automation handle this example? *SHELXT*, starting only with the assumption of monoclinic Laue symmetry and no prior stipulation of the space group, proposes two possible solutions: an ordered structure in *P*2_1_ is the clear favourite using default criteria in the program, while a disordered structure in *P*2_1_/*n* is feasible but less good. Subjection of the final *P*2_1_ structure to *PLATON*’s ADDSYM routine gives a 90% fit for an inversion centre and 95% for a glide plane and a suggestion of *P*2_1_/*n*, with the oxirane ring as the symmetry-breaking exception.

## A tougher case with an unexpected chemical structure   

**Figure 5:**
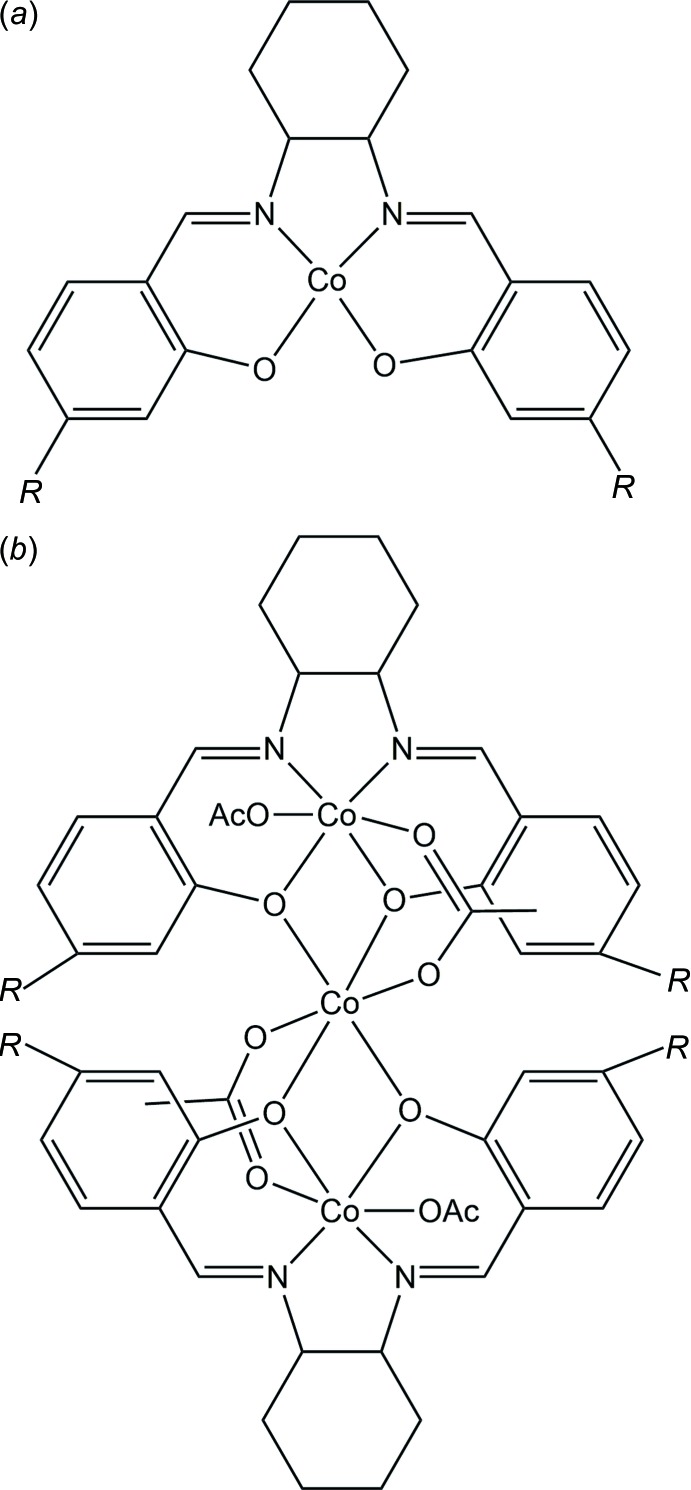
The originally proposed and experimentally determined chemical structures of the third example, a cobalt salen complex.

This structure has also been published as part of an investigation of coordinated salicylaldimine (salen) ligands (Achard *et al.*, 2012 ▸). Originally proposed by the chemists responsible for its synthesis as a mononuclear four-coordinate complex of cobalt(II) with a single salen ligand (Fig. 5 ▸ *a*), it was identified crystallographically as a trinuclear mixed cobalt(II/III) complex in which two oxidized mol­ecules of the proposed compound are linked by a Co^II^(acetate)_2_ unit and each carries an additional acetate ligand, all metal centres having octa­hedral coordination (Fig. 5 ▸ *b*).

This was another SRS-investigated sample, with low-temperature data measured this time shortly before final closure of the facility in 2008, Station 9.8 having been upgraded in the me­antime to use a Bruker *APEXII* diffractometer/detector system. The triclinic unit cell would accommodate two mol­ecules of the proposed mononuclear structure, though there is actually just one mol­ecule of the correct compound. Intensity statistics were unhelpful, the mean |*E* ^2^ − 1| lying between typical centric and acentric values, but this is not unknown for such metal complexes. A racemic structure with *Z* = 2 and both enanti­omers of the expected mol­ecule was a reasonable starting assumption.

No immediate solution was given by the routine direct methods procedures of *SHELXS*. The structure could be solved with non-default parameters, and from inter­pretation of the Patterson function (another example of crystallographic expertise that is widely unknown these days), whereupon the trinuclear formulation was recognized, with the central Co atom lying on an inversion centre in *P* 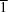 with *Z* = 1, *Z*′ = ½.

*SHELXL* (the 1997 version at the time) was used for refinement, which appeared to have no significant problems. With anisotropic displacement parameters and the inclusion of riding isotropic H atoms, the conventional *R* factor was around 0.09. Four atoms, all in the long alkyl chains, were flagged as ‘may be split’ because of high anisotropy, a feature often found in such groups. The six-membered ring in the ligand looked reasonable except that it was essentially planar and thus apparently aromatic instead of aliphatic, possibly an error in the chemical structure diagram supplied with the sample. There were no significant *checkCIF* alerts except for indications of unresolved disorder in the substituent chains as already noted. The structural model could be improved somewhat with some chain disorder modelling.

This structure is, however, wrong. Closer inspection shows that the displacement ellipsoids for the ring C atoms are all elongated perpendicular to the ring, although not enough to trigger any ‘may be split’ warnings. Chemists insisted that the rings are cyclo­hexane rather than benzene and should, therefore, be chair-shaped instead of planar. The single ring in the asymmetric unit can be resolved into two disordered, overlapping cyclo­hexane chair conformations, giving a stable improved refinement.

This, too, is still wrong. Further consultation with the chemists revealed that the ligand used in the synthesis was enanti­opure, with *R* absolute configuration at both chiral centres; this information had not originally been provided, and it is key to a solution of the problem. The crystal structure, assuming racemization has not occurred at any point, cannot be centrosymmetric, and the true space group must be *P*1 with the entire mol­ecule as the asymmetric unit. This removes the need for a disorder model for the rings, which are, in fact, fully ordered, with an appropriate model being derived by manual selection from the apparent disorder. Without inversion symmetry, the chain substituents also behave better in the refinement, the previously identified disorder disappearing; there may be minor disorder in these groups, but it has not been resolved and is adequately modelled by relatively large but sensible anisotropic displacement parameters.

**Figure 6:**
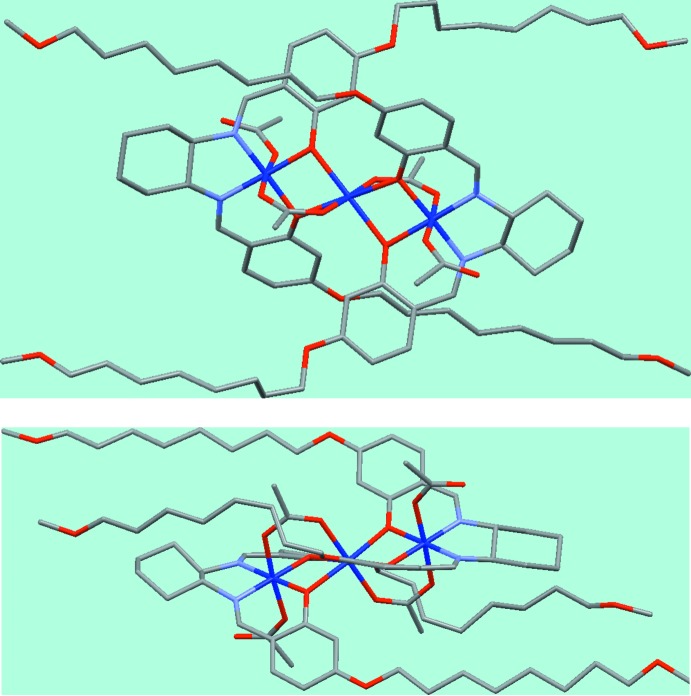
Two views (without H atoms) of the mol­ecule of the cobalt salen complex.

The final conventional *R* factor for the chiral *P*1 structure is 0.081; this is only a little lower than for the incorrect centrosymmetric structure, but that is because the deviations from inversion symmetry are very small, being restricted mainly to just a few light atoms. Because of this high degree of pseudo-symmetry, similarity restraints were required on the geometry and displacement parameters of the ligand atoms. The result is, however, entirely convincing and reliable, and is fully consistent with the known stereochemical mol­ecular properties of the sample. Two views of the mol­ecule, without H atoms, are shown in Fig. 6 ▸, in which the very close approximation to inversion symmetry apart from the ligand rings can be seen.

With hindsight, in 2019, the *P*1 structure is the preferred solution from *SHELXT*, the *P* 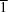 structure having a somewhat poorer Combined Figure of Merit. *PLATON*, however, still suggests *P* 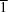 with a 100% fit within default tolerances. It may be that *SHELXT* is so successful in choosing the correct space group in such cases partly because it bases its decisions on examination of phase relationships between potentially symmetry-equivalent reflections in reciprocal space (primary data) rather than looking for evidence of symmetry operations between atoms in direct space (derived secondary data); perhaps the reciprocal space approach provides better discrimination.

## Conclusions and personal reflections   

The examples given here are just three selected from a lifetime’s crystallographic research that has included much investigation of structures that would yield readily to modern automatic procedures but also numerous cases that are not straightforward. As long ago as 1986, when conference posters were still something of a novelty, I won a prize for a poster entitled ‘Avoiding the Swamps and Marshes’ at the BCA Spring Meeting in York, describing a range of structures that had presented obstacles including aspects of symmetry. Other challenges to the ‘GO Button’ approach encountered over the years have included atom-type assignments, tricky disorder situations, unusual mol­ecular geometry (is it genuine?), and the correct treatment of hydrogen atoms. Here the focus has been on symmetry, partly because it is a subject fundamental to crystallography, and yet cohorts of students have questioned the importance of knowing and understanding it, especially when so many of its aspects can apparently be handled by ‘black box’ software. It is also a topic I have had the privilege of teaching on intensive crystallography courses in the UK and elsewhere for many years and have experienced the pleasure of seeing comprehension and relevance dawn on faces and in minds of developing young researchers.

The moral of the story is that crystallographers, if they are to avoid making serious and embarrassing mistakes, really do need to know some basic symmetry information and how to apply it, including the meaning and relationships of various concepts and terms.

In conclusion, the advice is:

do not trust entirely in automation;

do not rely unthinkingly on validation procedures;

take note of all relevant available information.

## Figures and Tables

**Table 1:** Comparison of triclinic and monoclinic refinements

	Triclinic *P* 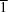	Monoclinic *P*2_1_/*n*
*Z*′	2	1
*R*_int_^*a*^	0.053	0.132
*R*_w_ (on *F* ^2^, all data)^*b*^	0.133	0.145
*R* (on *F*, data with *F* ^2^ > 2σ)^*c*^	0.058	0.065
Max *K* in analysis of variance^*d*^	4.01	7.47
Max difference peak (e Å^−3^)	1.32	0.83

Notes: (*a*) 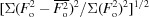 for equivalent reflections; (*b*) 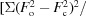 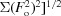 for all unique measured data; (*c*) 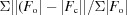 for unique reflections with 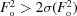; (*d*) 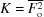/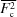 for batches of data in ranges of intensity and resolution.

**Table 2:** Comparison of triclinic and monoclinic refinements, including twinning

	Triclinic *P* 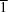	Monoclinic *P*2_1_/*n*	Triclinic *P* 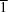 twinned
*Z*′	2	1	2
*R*_int_	0.053	0.132	0.053
*R*_w_ (on *F* ^2^, all data)	0.133	0.145	0.098
*R* (on *F*, data with *F* ^2^ > 2σ)	0.058	0.065	0.043
Max *K* in analysis of variance	4.01	7.47	1.09
Max difference peak (e Å^−3^)	1.32	0.83	1.13
Twin fraction			0.832 (2):0.168 (2)
